# Dislocation Arthropathy of the Shoulder

**DOI:** 10.3390/jcm11072019

**Published:** 2022-04-04

**Authors:** Ismael Coifman, Ulrich H. Brunner, Markus Scheibel

**Affiliations:** 1 Department of Orthopaedic Surgery and Traumatology, IIS-Fundación Jiménez Díaz, Universidad Autónoma de Madrid (UAM), 28049 Madrid, Spain; ismael.coifman@quironsalud.es; 2 Department for Traumatology and Orthopaedics, Krankenhaus Agatharied, 83734 Hausham, Germany; u.brunner@ugdb.org; 3 Department of Shoulder and Elbow Surgery, Schulthess Clinic Zurich, 8008 Zurich, Switzerland; 4 Center for Musculoskeletal Surgery, Campus Virchow, Charité-Universitaetsmedizin Berlin, 10117 Berlin, Germany

**Keywords:** glenohumeral osteoarthrosis, shoulder dislocation, shoulder instability, dislocation arthropathy, arthroplasty

## Abstract

Glenohumeral osteoarthrosis (OA) may develop after primary, recurrent shoulder dislocation or instability surgery. The incidence is reported from 12 to 62%, depending on different risk factors. The risk of severe OA of the shoulder following dislocation is 10 to 20 times greater than the average population. Risk factors include the patient’s age at the first episode of instability or instability surgery, bony lesions, and rotator cuff tears. For mild stages of OA, arthroscopic removal of intraarticular material, arthroscopic debridement, or arthroscopic arthrolysis of an internal rotation contracture might be sufficient. For severe stages, mobilization of the internal rotation contracture and arthroplasty is indicated. With an intact rotator cuff and without a bone graft, results for anatomical shoulder arthroplasty are comparable to those following primary OA. With a bone graft at the glenoidal side, the risk for implant loosening is ten times greater. For the functional outcome, the quality of the rotator cuff is more predictive than the type of the previous surgery or the preoperative external rotation contracture. Reverse shoulder arthroplasty could be justified due to the higher rate of complications and revisions of non-constrained anatomic shoulder arthroplasties reported. Satisfactory clinical and radiological results have been published with mid to long term data now available.

## 1. Introduction

Instability-mediated OA is a particular degenerative joint disease after a primary or recurrent dislocation or after instability surgery. In 1982 Neer [1] reported on 26 patients that had been treated with total shoulder arthroplasty due to severe OA after recurrent instability and anterior or posterior surgical stabilization in most cases: “recurrent dislocations and preceding surgery have tensed the capsule and thereby caused a fixed subluxation in the opposite direction of the instability”. Since then, it has been discussed whether this unique type of OA is caused by the primary dislocation and thereby is predetermined, or recurrent dislocations, concomitant fractures, other risk factors, and different surgical therapies themselves worsen the prognosis or even cause it. This review clinical article deals with the following aspects: physical examination, diagnostic radiology, causes, prognostic factors, and treatments options and their results so far.

## 2. Physical Examinations

Typical examination finding in patients with recurrent instability and OA is an increasing loss of range of motion, particularly a restriction of external rotation. Rosenberg et al. reported a mean limitation in external rotation of 18° with the arm at the side and 15° in 90° abduction in patients evaluated 15 years after open Bankart reconstruction [2]. Pelet et al. found a mean loss of external rotation of 24° in their retrospective 29-year follow-up study following open Bankart repair [3]. Oh et al. evaluated the association between shoulder OA and functional results as determined by the DASH score, which was significantly increased according to the severity of shoulder OA [4]. As a matter of course, severe pain and joint crepitation are commonly found in patients suffering from OA [5]. A rotator cuff examination is essential to rule out tears and insufficiencies. Particular attention should be paid to the subscapularis muscle after the anterior approach, which involves its detachment if the patient presents with pain, recurrent instability, weakness in internal rotation, and increased external rotation. An essential examination should include a lift-off test, internal rotation lag sign, modified belly press test, and belly-off sign [6].

## 3. Diagnostic Radiology

**Figure 1:**
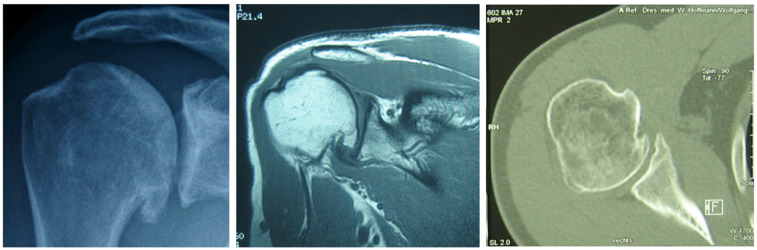
Imaging methods for evaluating dislocation arthropathy. Examples of conventional radiographs, MRI, and CT scan showing OA.

Conventional radiographs usually confirm the diagnosis of OA in advanced stages or provide essential hints for differential diagnosis. The typical findings of OA are narrowing of joint space as an indirect sign of reduction of cartilage, subchondral sclerosis representing an adaptation reaction of the bone, and metaplastic responses known as osteophytes [7]. The extent of these changes is underestimated by plain-film radiography [8]. Computer tomography (CT) could finally increase the accuracy in diagnosis and the prevalence of OA essentially (Figure 1). In 282 patients with unilateral instability without surgery, the rate of OA was 11.3% in conventional radiographic imaging and 31.2% when CT was used. Even small osteophytes can be detected. CT can also detect the loss of the anterior and posterior part of the joint gap when it remains almost unaltered in conventional radiographic ap-projection [9]. Ogawa et al. [10] reviewed 167 joints of 163 patients undergoing the open Bankart procedure. Preoperative CT showed OA in 44 shoulders (26.3%), among which 12 shoulders (7.2%) showed OA on the preoperative radiographs. Preoperative CT-proven OA in 20 shoulders never became visible on postoperative radiographs. Recent three-dimensional computed tomography (3D CT) trends could better define the relationship between the humeral head and the glenoid [11]. Posterior wear after index surgery should be analyzed if the progression of OA is recognized to define new treatment options [12]. Ultrasound is the method to depict intra-articular effusion at early stages. Osteophytes or the degree of synovitis are also visible. Subchondral lesions, changes in cartilage volume, and concomitant soft-tissue alterations were detected earlier using MRI [7,9]. MRI also enables semi-quantitative analysis of the postoperative changes of the subscapularis muscle. The results provide indications of the causes of the clinical dysfunction of the subscapularis musculotendinous unit after open shoulder stabilization [13].

## 4. Classification

In 1983 Samilson and Prieto [14] reported 74 patients with OA after multiple dislocations or surgical stabilization. They defined the term Dislocation Arthropathy and the Samilson and Prieto Classification (SPC) and suggested three stages based on anteroposterior radiographic images:

- Mild OA: Osteophytes on the lower humeral head or the lower glenoid rim <3 mm
- Moderate OA: Osteophytes on the lower humeral head or the lower glenoid rim between 3–7 mm
- Severe OA: Osteophytes on the lower humeral head or the lower glenoid rim larger than 7 mm with slimming of the joint gap and sclerosis

**Figure 2:**
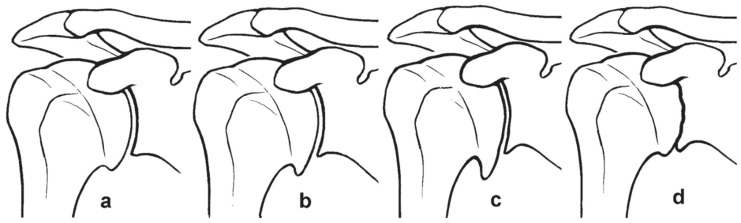
Image modified from Buscayret et al. [15]. (**a**): humeral osteophyte <3 mm. (**b**): humeral osteophyte >3 mm and <8 mm. (**c**): humeral osteophyte >8 mm. (**d**): obliteration of glenohumeral joint space.

To increase the classification accuracy according to Samilson and Prieto, Buscayret et al. suggested subdividing the severe OA stage into two stages: one with humeral osteophytes above 8 mm and a last one with the loss of the joint gap (Figure 2) [15].

The Samilson and Prieto classification is radiographic, but can it draw clinical conclusions? For example, Kircher et al. correlated pain, active and passive range of motion with OA graded according to Samilson and Prieto, finding the primary clinical feature, pain, as the main indication for surgery, not related to radiological parameters. In addition, the increasing size of the caudal humeral osteophyte was associated with a decreased functional status in all planes [16].

SPC is based only on 2D examination. Recently, Link et al. found no correlation between SPC and Walch classification for primary OA. Therefore, understanding glenoid morphology in the axial plane is mandatory in the final stage of OA for correct implant selection. However, no validated classification has been published to assess Dislocation Arthropathy in the axial plane [17].

## 5. Causes and Prognostic Factors

Since the first descriptions of OA after instability were carried out on patient groups that underwent surgery in most cases, it was presumed that OA results from the surgery itself [18].

### 5.1. Development of OA after Non-Operative Management of Shoulder Instability

Hovelius found that with a follow-up of 225 patients with first-time dislocations after 25 years, the spontaneous process after first-time dislocation accompanies OA development [19]. In a prospective study of patients with first-time dislocation after conservative therapy, 16.1% of 106 patients with a single dislocation without recurrence after ten years developed OA. With and without recurrence and operation, 11% of 208 shoulders had slight and 9% moderate or severe OA. The interesting point is that the shoulders with only one recurrence had similar rates of OA to those with recurrent dislocations or operations [20].

Hovelius [19], and Singer [21], conclude that the primary dislocation introduces the development of the OA and that later recurrences are in this regard of minor importance. Ogawa et al., however, found that the number of the dislocations/subluxations was significantly different between shoulders with and without OA [9].

Buscayret et al. analyzed the pre and postoperative radiologic processes of 570 patients that had undergone shoulder stabilization [15]. They found five factors with statistically significant influence on the development of a preoperative OA without operation: The age at the first-time dislocation and at the time of the operation, in each case with higher risk at higher age; bony defects at the front lower glenoid or at the humeral head as well as a rotator cuff tear. Kraus et al. evaluated the results of conservative treatment of acute anteroinferior glenoid fractures [22]. Intra-articular step-off amounted to 6 mm (mean 2 mm); nevertheless, no significant increase in the OA rate could be found after a mean follow-up of 26.4 months. Marquiera et al. evaluated 14 patients with large Bankart fractures (>5 mm) and dislocation >2 mm that underwent conservative treatment [23]. After a mean follow-up of 5.6 years, every shoulder was stable. Only two patients showed mild and one patient moderate radiographic signs of arthrosis. Finally, Weisser et al. recently published excellent results of nonoperative treatment of anterior glenoid rim fractures after primary traumatic anterior shoulder dislocation. In the cohort of 30 patients with a >5 mm anterior glenoid rim fracture, functional outcome was reported as excellent with a low rate of recurrent instability (3%) and a low rate of new-onset OA (23%). To achieve these outcomes well centered post-reduction humeral head was mandatory. Anterior subluxation after reduction might develop in recurrent instability and OA, and should be considered a contraindication for nonoperative treatment [23,24].

The risk of developing a severe OA for individuals that suffered a shoulder dislocation is 10–20 fold increased [25]. The risk factors after conservative and surgical therapy are partially congruent (age, extended time until operation, bony defects, alcohol, smoker, hyperlaxity, high BMI, and increased age at initial instability event) [15,26,27,28].

### 5.2. OA after Surgical Stabilization

The incidence of OA after anterior surgical stabilization is stated between 12–62% [2,4,29,30]. In a prospective study with 41 patients that had at least two anterior dislocations and underwent arthroscopic transglenoidal suture, 12% showed radiographic changes after a follow-up of 52 months. There was a significant correlation between these changes and a worse clinical outcome. Patients with Bankart and Hill–Sachs lesions or other bony alterations on the preoperative images presented with a significantly worse functional outcome [4].

In a retrospective study, 30 of 39 patients that underwent open Bankart reconstruction could be examined after a mean follow-up of 29 years. Five patients were treated with total shoulder arthroplasty, and seven presented with radiographic signs of OA. Overall, the rate of OA was 40%. The authors, therefore, concluded that even though satisfying long-term results could be attained, the development of OA could not be stopped by surgery [3].

In another retrospective study after open Bankart reconstruction 33 of 53 shoulders could be evaluated after 15 years; 87% presented with no or minor radiographic signs of OA, 14 patients with minimal, and one patient with severe signs of OA. A significant correlation between the radiographic degenerative alterations and limitation of external rotation in 90° abduction depending on the time of follow-up could be shown. An influence of the limited external rotation in developing arthrosis was discussed but could not be proved [2]. In 2010, Ogawa et al. reviewed 163 patients undergoing the open Bankart procedure, finding that the development and progression of OA cannot be prevented by surgical intervention [10]. Most postoperatively detected OA developed already before surgery; nevertheless, the progression of postoperative OA was prolonged. Recent studies with over 20 years of follow-up still report satisfying outcomes. Moroder et al., with a mean 22-year follow-up after open Bankart repair in 26 patients, reported good clinical outcomes with minimal loss in the range of motion [31]. However, OA was found in up to 50% of patients and was associated with loss of external rotation, raising the question of whether the loss of external rotation was caused by OA or by overtightening the anterior capsule.

After Latarjet stabilization, 56 of 95 patients could be evaluated after a mean follow-up of 14.3 years. Three factors could be identified to be relevant for the development of postoperative OA: rotator cuff lesions, intra- or postoperative complications, and positioning of the coracoid to lateral. This last one is the most important prognostic factor. It was discussed that even though the rate of OA is quite high, stage I OA seems not to influence the postoperative outcome even after ten years [29]. Mizuno et al. conducted a retrospective review of 68 open Latarjet patients with a mean follow-up of 20 years. Of the 60 shoulders without OA preoperatively, 12 developed OA at final follow-up [32]. Overall, postoperative OA was mild, finding stage 1 in 14.7%, stage 2 in 5.9%, and stage 3 in 8.8% of patients. On the other hand, Gordins et al. report 65% of OA in 31 patients after 33 years of follow-up open Latarjet [33]. However, the technique implemented was the one described by May, and all patients were operated on before the modified Latarjet technique by Patte et al. [34]. Coracoid dimensions and standing up “May coracoid transfer” might influence these OA outcomes.

Comparing the rate of OA after Bankart and Bristow–Latarjet procedures after ten years of follow up Hovelius found a higher incidence after the Bankart procedure (16 of 26) than after the Bristow–Latarjet procedure (9 of 30). A recent meta-analysis suggests that the Latarjet procedure has a lower OA degree than other treatments, including non-operative treatment [35].

After glenoid reconstruction of significant bony defects using a J-graft, most relevant studies showed that there was no significant correlation between the number of dislocations and the rate of OA and that a significant influence of the performed surgery could not be found [26]. A recent follow-up of the cohort published by Moroder et al. shows excellent results regarding stability and function after a mean follow-up of 18 years [36]. However, OA was present in 74% of the patients now. Therefore, the development of dislocation arthropathy may not be prevented by this procedure.

Of 34 patients that underwent Weber-osteotomy, only four (9%) had no OA, nine (26%) had been treated with total shoulder arthroplasty. The increased internal rotation and the degree of arthrosis were statistically significant [37].

The rate of OA after the Eden–Hybinette procedure is always mentioned to be one of the highest [38]. In a retrospective study including 74 shoulders with a mean follow-up of 29 years after the Eden-Hybinett procedure, a recurrence occurred in 15 cases (20%) and OA in 35 cases (47%).

The ages at the time of the primary dislocation, surgery, and follow-up were mentioned as risk factors. Shoulders with signs of OA showed significant limitations of external rotation, even though most of them were subjectively satisfied [39]. Comparing 2- and 5-years follow-ups, the degree of limitation in the external rotation was not correlated significantly to the rate of OA. The rate of arthrosis was higher after primary dislocation at a higher age (above 23) [40]. Buscaryet et al. showed in 570 patients after surgical stabilization that lower degrees of OA remain without progress more often than higher degrees; 19.9% of the patients who had no preoperative signs of OA developed postoperative OA [15]. The lengths of follow-up and the number of preoperative dislocations were found as risk factors. The time to surgery, the degree of instability (luxation or subluxation), the level of sportive activity, and especially the type of surgery were found to have no significant correlation with the development of OA. Therefore, there was no difference found between the Latarjet-procedure and soft-tissue techniques. When comparing the three groups with equal follow-up, no significant differences could be found concerning the rate of OA.

After arthroscopic stabilization, there was a lower rate of OA but a lower time of follow-up [15]. Other authors found similar rates of OA both in open and arthroscopic procedures [7]. Boileau et al. reported an increase in glenohumeral OA incidence from 4% preoperatively to 17% postoperatively after arthroscopic stabilization [41]. Meantime, a couple of literature reports deal with the long-term appearance of OA after arthroscopic Bankart repair. Castagna et al. found mild (29%) to moderate (10%) arthrosis after a minimum of 10 years after arthroscopic Bankart repair, but degenerative changes of the glenohumeral joint had no significant effect on the clinical outcomes [42]. Kavaja et al. examined the radiologic and clinical occurrence of glenohumeral OA 13 years after arthroscopic Bankart repair [43]. OA was diagnosed radiologically in 68 percent but rarely caused subjective symptoms. Franceschi et al. found OA in 21.8% of the patients with no preoperative degenerative changes eight years after arthroscopic Bankart repair [44]. Finally, the latest arthroscopic Bankart repair cohort published by Plath et al. reports 69% of OA over a hundred patients with a mean follow-up of 13 years [45].

**Figure 3:**
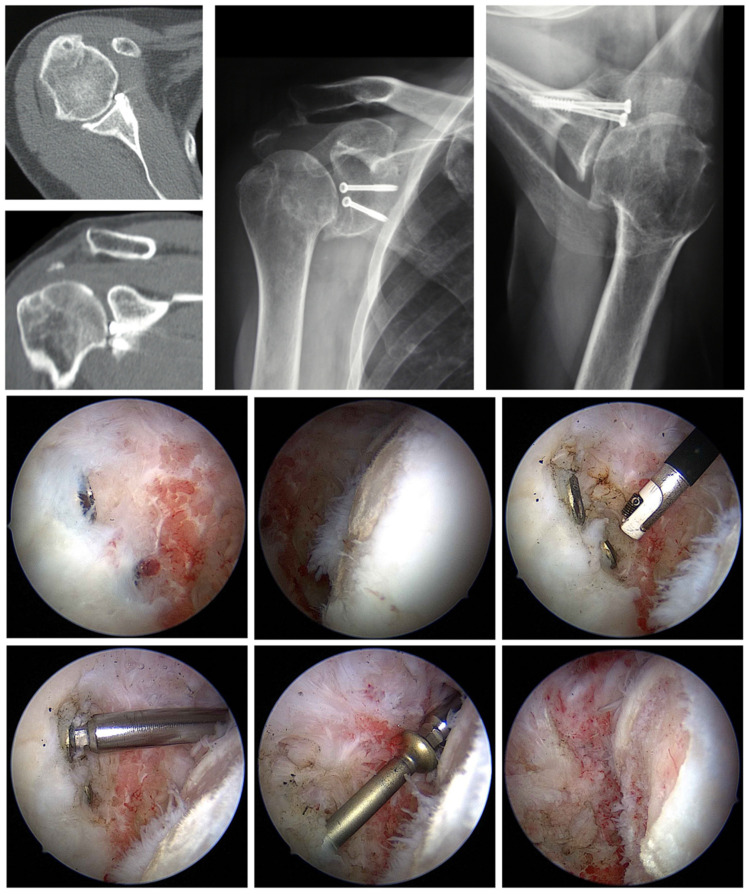
Details of proud screws after glenoid fracture surgery removed arthroscopically. **First row**: radiologic studies showing instability surgery performed and proud implants. **Second row**: Images of a humeral cartilage defect and debridement necessary to expose implants. **Third row**: Arthroscopic screws removal.

These studies show that postoperative OA in different degrees occurs, both in open and arthroscopic procedures. As risk factors in developing postoperative OA, bony lesions (Bankart and Hill–Sachs lesions), lengths of follow-up, concomitant lesions of the rotator cuff, intra- and postoperative complications, positioning of the coracoid to lateral, higher age at primary dislocation or surgery, and a long time to surgery are mentioned. In addition, loose or proud metal pieces (screws, staples) (Figure 3) could cause a progressive OA quickly [46]. Yeh and Kharrazi report a rare but dramatic complication following shoulder arthroscopy: post arthroscopic glenohumeral chondrolysis [47]. The articular cartilage undergoes rapid degenerative changes shortly after arthroscopic surgery. Although the etiology of post arthroscopic glenohumeral chondrolysis is not yet fully understood, the pathophysiology is likely multifactorial.

After arthroscopic stabilization using screws positioned at the glenoidal rim, Tauber et al. found only one case in 10 cases of material impingement that made the removal necessary after two years [48]. Experimental studies show that a loss of the anteroinferior labrum reduces the contact area by 7–15% and increases the contact pressure by 8–20%, concerning the anteroinferior part of the glenoid even at 53%. A bony loss of 30% of the glenoid diameter increases the contact pressure at the anteroinferior part even at 300–400% [49]. Whether such a loss or step-off formation is relevant for instability and development of arthrosis seems to be dependent on a centered or decentered humeral head.

### 5.3. Capsulorrhaphy Arthropathy as an Own Entity?

Matsen et al. defined the term capsulorrhaphy arthropathy for patients who develop OA due to too strongly strained anterior capsules [50]. The strong harnessing of the anterior soft tissues, e.g., a Putti–Platt, or a too strongly strained Bankart operation, leads to compression and intensified shearing stresses on the joint surface that increase if the patient goes into external rotation. It is postulated that this mechanism develops in all operations where the external rotation is excessively limited [51].

**Figure 4:**
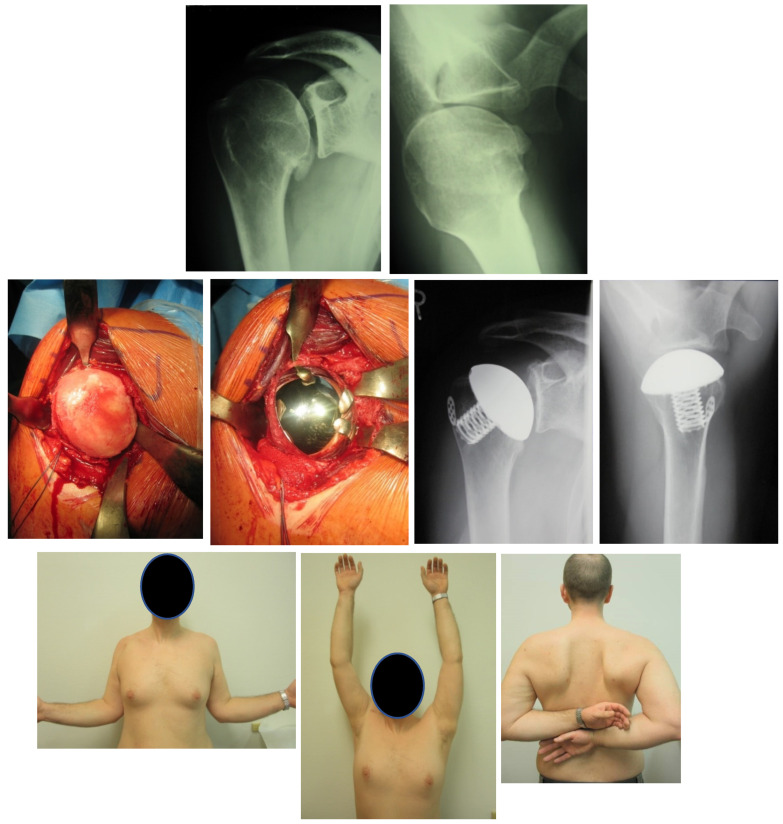
43 year-old patient treated with hemiprosthesis after capsulorrhaphy arthropathy subsequent to open instability repair. **First row**: preoperative radiographs. **Second row**: intraoperative pictures of hemiprosthesis implant and postoperative radiographs. **Third row**: Physical examination and shoulder function at final follow-up.

Biomechanical and anatomical studies today offer evidence that a non-anatomical strain of the anterior capsule leads to an increase in posterior joint pressure, posteroinferior subluxation of the humeral head, and thus pain and the development of arthrosis [52]. In a cadaveric comparison of a front capsule strain with an anteroinferior capsule shift, it could be shown that during the strain of the front capsule the stability decreases and the external rotation and elevation are limited. That larger shear joint forces are necessary to reach the maximum elevation. In contrast, the anteroinferior capsule shift improves stability without limiting the external rotation or elevation [53] (Figure 4).

Gerber and Werner experimentally showed the effect of selective capsulorrhaphy on the translation and the passive range of motion [54,55].

On the one hand, these studies document that capsulorrhaphy arthropathy is biomechanically justified and permits, on the other hand, developing more anatomical stabilization operations. In retrospective studies, a decreased external rotation was connected with an increased rate of OA; whether this was the cause or the effect could not be clarified [15].

## 6. Treatment Options and Results

### 6.1. Nonoperative Treatment

Non-operative treatment of shoulder dislocation arthropathy should be the first step of management. Classic studies have shown similar OA proportions between non-operative and operative treatment at any point of follow up [35]. There is no evidence of significant benefit in using non-steroidal anti-inflammatory drugs (oral or topic) to treat shoulder pain. Improvements could be found with oral prednisolone, but side effects should be taken into special consideration when using these medications. Intraarticular corticosteroids or hyaluronic acid are among the most popular nonoperative treatments for glenohumeral OA. While both have demonstrated sustained pain relief, difficulty in accurately administering them in the glenohumeral joint without ultrasound assistance has been pointed out. There are no efficacy studies regarding physical therapy as an isolated treatment. Several multimodal therapy plans have proven sustained improvements in pain and function [56].

### 6.2. Removal of Foreign Material

Metal anchors that contact the joint surface will lead to a progressive OA in the shortest time. Pain or crepitation after shoulder stabilization should, therefore, be clarified. The positions of possible metal anchors can be retraced in a thin slice CT. Without the slightest doubt, a revision arthroscopy and the removal of the anchors are necessary. The knowledge of the kind of brought-in anchors is vital to providing the right removal instrument. To approach the anchor in its centerline, percutaneous instrumenting can be helpful. Metal portions that are not visible in the joint at first sight could be covered by only a thin layer of soft tissue and should, therefore, be removed.

Implants for shoulder stabilization have evolved to suture anchors manufactured of various materials, including metal, poly-L-lactic acid, PEEK (polyether ether ketone), and all sutures. “Anchor arthropathy” could be defined as an own entity after stabilization surgery. Early-onset of pain and stiffness, usually before 10 months after index surgery, could be found. Waltz et al. found advanced imaging, such as MRI unreliable to confirm proud implants or prominent suture knots. Therefore, early arthroscopy to assess painful and stiff shoulders after instability repair should have a low threshold [57].

### 6.3. Arthroscopic Debridement and Arthrolysis

In the case of an early stage of OA arthroscopic, debridement with loose cartilage portions removal and partial synovectomy can improve functionality and relieve pain. The cause of arthritis, e.g., the eccentric load of the glenoid as its “engine”, is not resolved by this. An arthroscopic debridement can only help if a sufficient passive range of motion with the possibility of relieving after treatment is present. Removing the osteophytes, usually within the front lower range of the anatomical neck, is technically challenging. Millet’s CAM procedure was developed as a joint-preserving arthroscopic treatment approach for young, active patients with advanced shoulder OA [58]. Besides chondroplasty, synovectomy, loose body removal, and subacromial decompression, the CAM procedure also involves extensive capsular release to restore motion, humeral osteoplasty, and osteophyte excision to recontour the humeral head, restore abduction, and potentially decompress impingement on the axillary nerve; axillary nerve neurolysis when scarring is seen and biceps tenodesis when there is significant tenosynovitis, SLAP tear or a pulley lesion [59]. Arner et al. reported significant improvements in 38 patients after 10 years of follow-up of the CAM procedure. Humeral head flattening and severe joint incongruity were risk factors for CAM failure, although survivorship was 63% at a minimum 10-year follow-up [60]. A recent investigation from the same group found similar results after arthroplasty, whether a prior CAM procedure was performed before the prosthesis [61].

### 6.4. Arthroplasty

The problem with dislocation arthropathy is that these patients are younger than those with idiopathic OA and usually have substantial internal rotation contracture and posterior glenoid defects [62].

As with each OA, the preoperative clinical examination with determination of the rotation is essential. A limited external rotation is a prognostically negative criterion for the post-op result. The preoperative analysis of the glenoid constellation is often insufficient in the axial projection and is better investigated in the CT. The MRI is used to evaluate fatty atrophy and integrity of the subscapular muscle and the other portions of the rotator cuff.

During the approach, the mobilization with an anterior extension of the subscapular muscle is critical of particular importance. This can be achieved by completely separating the subscapular tendon and approximately 1 cm medial refixation. A medialization of around 1 cm corresponds to an external rotation gain of approximately 20°. In case of stronger contractions, a bifocal capsulotomy according to Habermeyer is preferred [63]. The incision begins at the rotator interval. After ligature of the anterior circumflex arteries and protection of the latissimus dorsi and teres major insertion, the subscapular muscle is wholly detached down to the metaphysis [64]. The medial mobilization behind the anterior margin of the coracobrachialis muscle is not recommended to preserve blood circulation and innervation and avoid secondary damage to the subscapular muscle.

**Figure 5:**
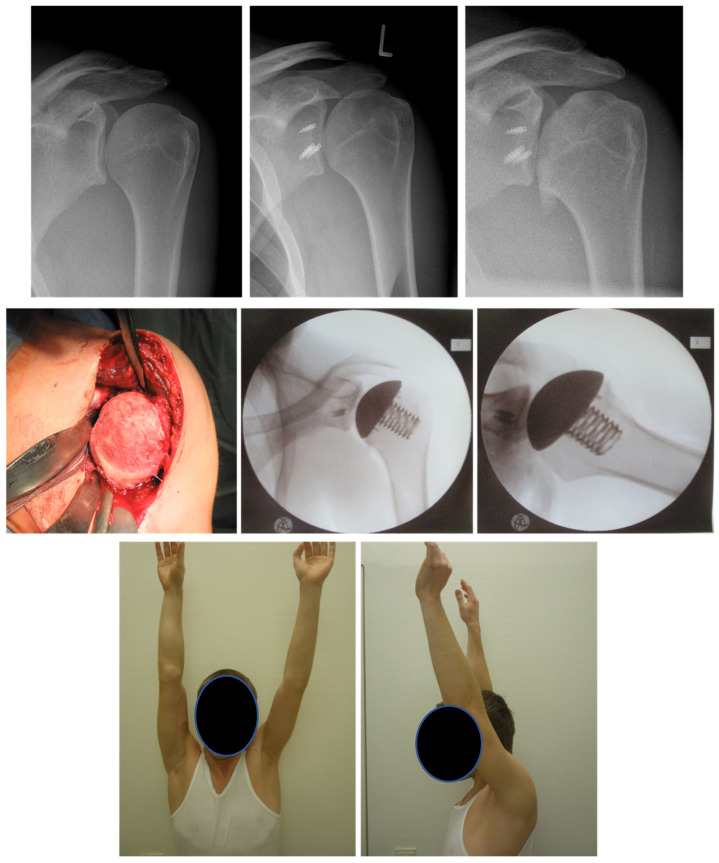
22-year-old patient with dislocation arthropathy after instability surgery treated with stemless prosthesis. **First row**: radiographs showing OA after shoulder instability surgery. **Second row**: intraoperative pictures of hemiprosthesis. **Third row**: Physical examination and shoulder function at final follow-up.

The replacement on the humeral side depends on the size of the defect. In younger patients, a cup, stemless or short stem/stem prostheses are possible (Figure 5). In the cup prosthesis, the bony defect should not exceed 30%. Recent studies report comparable short-term results between a combination of humeral surface replacement with cemented glenoid component and conventional total shoulder arthroplasty [65].

**Figure 6:**
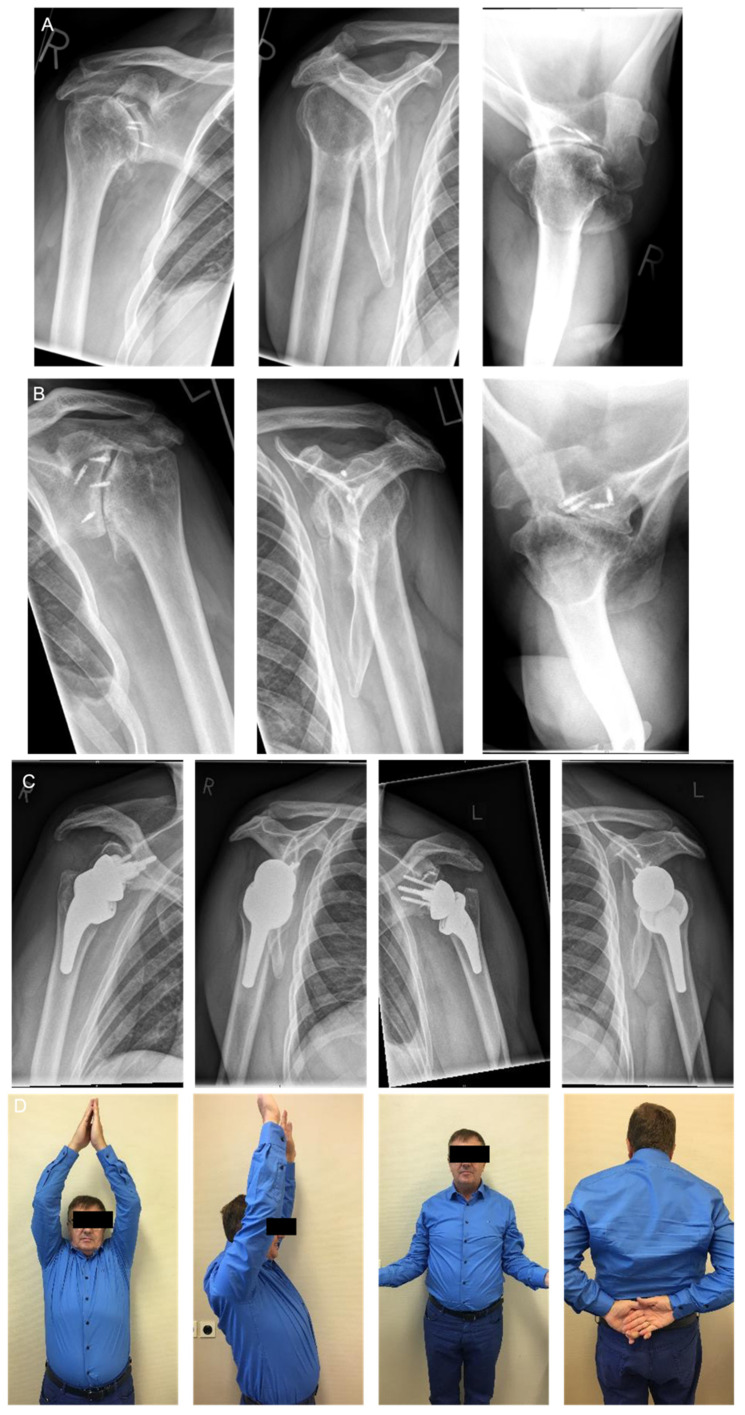
61-year-old male with bilateral dislocation arthropathy. (**A**): right shoulder dislocation arthropathy after instability surgery. (**B**): left shoulder dislocation arthropathy after instability surgery. (**C**): treatment with bilateral two surgeries Reverse Shoulder Arthroplasty using full wedge. (**D**): right side 12 months follow up and left side 6 months follow up clinical results.

Dorsal rolled out glenoids require excellent preoperative planning to define the glenoid form and version. After the good exposition, the axis and the glenoid center should be marked with, e.g., a K-wire to plan the correct inclination and version. The value of navigation still must be proven. In most cases, with sufficient bone substance, the higher edge of the glenoid is removed to create a correct version. In larger, usually posterior defects, a reconstruction by a bone graft (“contained defect”) or accumulation of an iliac crest graft (“non contained defect”) is necessary (Figure 6). Bone transplantation for re-establishment of the glenoid defects or correcting the version is already mentioned in small numbers by Neer [66] after introducing the unlinked prostheses. The simultaneous implantation of a cemented glenoid is problematic from a biological point of view. Here two-step procedures should be preferred. The fixation of anterior bone grafts is substantially more straightforward and unproblematic than that of posterior defects. The posterior bone grafts can be placed only sometimes from the anterior. If a dorsal defect without hold to the medial exists, the graft must be inserted from the posterior. The graft is fixed with two screws that should not affect the implantation of the glenoid (keel or pegs). If a strong posterior inclination of the glenoid is present and no sufficient correction of the version is possible, stability can be increased by adapting the version of the components against each other. If this is not possible, it is better to surrender the glenoidal component. In case of a simultaneous existing out-of-center rotator cuff lesion, the implantation of Reverse Shoulder Arthroplasty (RSA) is possible.

#### Results after Arthroplasty

Green and Norri [62] retrospectively evaluated 17 of 19 patients with shoulder prosthesis (15 TSA and two HSA) due to dislocation arthropathy (four Bristow, four Putti Platt, four Magnuson Stack, two Bankart, and four soft tissue operations) after 62 months; 94% had significant pain relief. Except for one, all patients received a better function. Subjectively, 16 patients judged the result as much better or better and one as worse. Three patients had to be revised.

Sperling et al. examined 31 patients (21 TSA, 10 HSA) retrospectively for at least two years and an average of 7 years postoperatively [67]. Pain, external rotation, and active abduction improved significantly without differences between HSA and TSA. The survival rate after two years was 97%, after five years 86%, and after ten years, only 61%. Nevertheless, 3/10 HSA and 8/21 TSA had to be revised.

Hill and Norris examined the results after bony glenoid reconstruction at five anterior and 12 posterior defects, five patients with arthrosis, three with capsulorrhaphy arthropathy, two with recurrent dislocations, and one after revision. All had a certain anterior or posterior instability preoperatively [68]. In 15 patients, a bone from the resected humeral head was used. The indication for transplantation of a bone graft existed, if the bone substance was not sufficient to correct the version (version >15°), to ensure the fixation of the glenoidal component (withdraw the keel when planning), or if via a version of the components no adjustment could be made. After correction, an average retroversion of 4° with an average correction angle of 33° could be seen. Three patients with graft failure (nonunion, dissolution, or graft dislocation) and five failures with glenoid revisions because of rotator cuff rupture, persisting instability, wrong component placement, or loosening of the transplant, showing unsatisfactory functional results. In 14 of 17 cases, the version and substance of the glenoid could be repaired. The patients without implant or transplant failure showed an apparent reduction in pain and good gain of function (AAE on average 107° (30–165°), i.e., satisfying functional results in nine of 17 patients. The study of Neer showed a lower failure rate (0 of 19) [66].

Primary glenoidal bone graft transplantation has a ten times higher risk of glenoidal failure than patients with primary implantation without bone transplantation. If the transplant heals sufficiently, there is no tendency for early loosening. The transplantation is suitable to lower the post-operational instability rate [68].

Matsoukis et al. examined two collectives in a multicentric study, one with and one without previous stabilization operation [69]. Twenty-eight patients without preceding operations had been seen at least for two years. One group sustained the first dislocation under and the other one over the age of 40 years. Below 40 years, the processes were longer, and there were numerous recurrences, but only one rotator cuff tear was found; 64% had an excellent or good result, similar to concentric osteoarthritis. The processes were short in the second group with patients older than 40 years. With seven rotator cuff tears, only 36% of them had an excellent or good result. Because of the rotator cuff tears, hemiprostheses had been implanted in most cases. The difference is probably due to the higher rate of rotator cuff tears.

In contrast to fatty degeneration of the rotator cuff, especially of the subscapularis muscle, the preceding operation and the preoperative external rotation did not influence the result. Altogether, prosthetics could achieve good results due to dislocation arthropathy after conservative and operational treatment. Significantly better results were shown after TSA than HSA. Adverse prognostic factors were a higher age at the initial dislocation and a rotator cuff tear. The previous surgery, e.g., bone block or soft part operation, was without significant influence (10 complications in 55 prostheses, three cases of glenoid loosening in connection with rotator cuff ruptures, four anterior instabilities, six revisions) [70]. Lehmann et al. report a significantly increased weighted average constant score following shoulder arthroplasty for OA caused by shoulder instability [63]. The authors found no significant difference between total shoulder replacements and hemiarthroplasty. Nevertheless, a relatively high rate of complications (40%) was revealed, with 20% requiring an operative revision.

Due to inconsistent results, surgeons are moving towards the implantation of RSA after dislocation arthropathy (Figure 6). There is a trend of positive results with these implants, yet follow-up is still relatively short. RSA has been used in recent years for patients with OA and rotator cuff deficiency after shoulder stabilization. Raiss et al. describe the results of 13 patients with a median follow-up of 3.5 years and a median age of 70 years that had at least one rotator cuff tendon tear in combination with an OA treated before for recurrent anterior shoulder instability. Constant score, shoulder flexion, and internal rotation significantly improved after RSA and were comparable with those of other studies reporting on the outcome of reverse shoulder arthroplasty for other conditions [71].

For Clavert et al., RSA is justified due to the higher rate of complications and revisions of non-constrained anatomic shoulder arthroplasties reported. In his cohort of 25 patients with a mean follow-up of 6.6 years, clinical results were comparable to other studies describing results of RSA, even in cases where bone grafting was mandatory [72]. Besides satisfactory clinical and radiological results have been published, and follow-up is still relatively short for this indication.

## 7. Conclusions

Dislocation arthropathy can be the consequence of an instability episode of the shoulder. The risk of developing OA after primary traumatic dislocation compared to the normal situation is increased up to 10–20-fold. The age at the initial dislocation, bony lesions at the glenoid, and the head or a rotator cuff tear increase the risk. The classification according to Samilson and Prieto is used. CT and MR tomographic diagnostics increase the genesis statement, the classification, and the therapeutic options. Rates of OA after stabilization range between 12 and 62%, whereby a safe designation of the operational procedures cannot be made. The Latarjet procedure seems to have a lower degree of OA than other treatments, even conservative treatments. Metal anchors and screws with joint contact lead to a rapidly progressive OA and must be removed, arthroscopically. In low-grade OA, arthroscopic debridement is helpful. Arthroscopic arthrolysis with capsulotomy can improve elevation and external rotation in cases of internal rotation contracture. If massive OA is present, prosthesis becomes inevitable. However, patients with instability arthropathy are mostly younger and suffer from a considerable internal rotation deficit and glenoid defects. The defect size determines the humeral replacement. Using Cup-prostheses, it should not exceed 30%. Results of total shoulder prosthesis are superior to those of hemiarthroplasty. Three-dimensional planning tools are becoming useful for correct implant selection. Results of RSA are promising; however, a longer follow-up is required. Significant glenoid defects need to be treated with bone grafting to provide stability. Nevertheless, bone grafting increases the risk of implant failure. The type of primary treatment and external rotation did not affect the prognosis of the prosthesis after glenohumeral stabilization, whereas fatty degeneration of the rotator cuff did.
